# Empagliflozin improves renal ischemia–reperfusion injury by reducing inflammation and enhancing mitochondrial fusion through AMPK–OPA1 pathway promotion

**DOI:** 10.1186/s11658-023-00457-6

**Published:** 2023-05-18

**Authors:** Wenbo Yang, Xiaoli Li, Liujie He, Shuyang Zhu, Shicong Lai, Xiaopeng Zhang, Zixiong Huang, Biyue Yu, Chunping Cui, Qiang Wang

**Affiliations:** 1 Department of Urology, Peking University People’s Hospital, Beijing, 100044 China; 2 Department of the Eighth Healthcare, the Second Medical Center & National Clinical Research Center for Geriatric Diseases, Chinese PLA General Hospital, Beijing, 100853 People’s Republic of China; 3 Naval Medical University, Shanghai, 200433 China; 4 School of Life Sciences, Hebei University, Baoding, 071002 Hebei China; 5 State Key Laboratory of Proteomics, National Center for Protein Sciences (Beijing), Beijing Institute of Lifeomics, Beijing, 100850 China

**Keywords:** Renal ischemia–reperfusion injury, Empagliflozin, SGLT2i, Inflammation, Mitochondrial dynamics, OPA1, AMPK signaling

## Abstract

**Background:**

Renal ischemia–reperfusion injury (IRI) is one reason for renal transplantation failure. Recent studies have shown that mitochondrial dynamics is closely related to IRI, and that inhibition or reversal of mitochondrial division protects organs against IRI. Optic atrophy protein 1 (OPA1), an important factor in mitochondrial fusion, has been shown to be upregulated by sodium-glucose cotransporter 2 inhibitor (SGLT2i). Also, the antiinflammatory effects of SGLT2i have been demonstrated in renal cells. Thus, we hypothesized that empagliflozin could prevent IRI through inhibiting mitochondrial division and reducing inflammation.

**Methods:**

Using hematoxylin–eosin staining, enzyme linked immunosorbent assay (ELISA), flow cytometry, immunofluorescent staining, terminal deoxynucleotidyl transferase (TdT)-mediated dUTP nick end labeling (TUNEL) staining, real-time PCR, RNA-sequencing, and western blot, we analyzed renal tubular tissue from in vivo and in vitro experiments.

**Results:**

Through animal experiments and sequencing analysis, we first confirmed the protection against IRI and the regulation of mitochondrial dynamics-related factors and inflammatory factors by empagliflozin pretreatment. Then, through hypoxia/reoxygenation (H/R) cellular experiments, we confirmed that empagliflozin could inhibit mitochondrial shortening and division and upregulate OPA1 in human renal tubular epithelial cell line (HK-2) cells. Subsequently, we knocked down OPA1, and mitochondrial division and shortening were observed, which could be alleviated by empagliflozin treatment. Combined with the previous results, we concluded that OPA1 downregulation leads to mitochondrial division and shortening, and empagliflozin can alleviate the condition by upregulating OPA1. We further explored the pathway through which empagliflozin functions. Related studies have shown the activation of AMPK pathway by empagliflozin and the close correlation between the AMPK pathway and OPA1. In our study, we blocked the AMPK pathway, and OPA1 upregulation by empagliflozin was not observed, thus demonstrating the dependence of empagliflozin on the AMPK pathway.

**Conclusion:**

The results indicated that empagliflozin could prevent or alleviate renal IRI through antiinflammatory effects and the AMPK–OPA1 pathway. Ischemia–reperfusion injury is an inevitable challenge in organ transplantation. It is necessary to develop a new therapeutic strategy for IRI prevention in addition to refining the transplantation process. In this study, we confirmed the preventive and protective effects of empagliflozin in renal ischemia–reperfusion injury. Based on these findings, empagliflozin is promising to be a preventive agent for renal ischemia–reperfusion injury and can be applied for preemptive administration in kidney transplantation.

**Supplementary Information:**

The online version contains supplementary material available at 10.1186/s11658-023-00457-6.

## Background

Ischemia–reperfusion injury (IRI) is one of the main clinical causes of early renal dysfunction after renal transplantation [1]. Due to the anatomical characteristics of the kidney, renal tubular epithelial cells are extremely sensitive to IRI. Severe IRI can lead to delayed graft function (DGF) with acute tubular necrosis as the main pathological manifestation, which can induce acute and chronic rejection immune response and reduce graft survival rate [2].

Both innate and adaptive immune systems are involved in the inflammatory immune process leading to kidney injury after IRI [3]. Proinflammatory injury-related molecular patterns, hypoxia-inducible factors, adhesion molecules, renal vascular endothelial dysfunction, chemokines, cytokines and Toll-like receptors are all involved in activating and recruiting immune cells into the injured kidney. Immune cells, such as neutrophils, dendritic cells, macrophages, and lymphocytes are involved in the pathogenesis of renal injury after IRI [4, 5]. Therefore, reducing the sterile inflammation that develops after IRI is of vital significance for renal protection.

In addition to causing an inflammatory response in the extracellular environment of the kidney, IRI also disrupts the homeostasis of the intracellular environment. Mitochondrial dynamics play an essential role in maintaining the structural integrity of mitochondria and adapting to different cellular biological functions. Notably, mitochondrial dynamics have been shown to exert critical effects on cell survival under stress conditions such as IRI [6, 7]. IRI induces mitochondrial division to compensate for the lack of energy metabolism [8], but excessive mitochondrial division eventually mediates cell death [9]. It is now believed that intervention or reversal of mitochondrial division is one of the key means to avoid IRI. This protective effect has now been demonstrated in models of acute IRI in liver cells and cardiac myocytes [10, 11]. Moreover, in a previous study by our group, we found that promoting mitochondrial fusion reduced renal cell injury and apoptosis during IRI, suggesting that the regulation of mitochondrial dynamics plays an important role in the development of renal IRI [12].

Optic atrophy protein 1 (OPA1) has been found to be a major fusion factor regulating the mitochondrial fusion process [13, 14]. Several studies have shown that downregulation of OPA1 expression can cause increased sensitivity of mammalian cells to exogenous proapoptotic stimuli and promote the development of apoptosis [15, 16]. Increased OPA1 expression effectively enhances mitochondrial fusion, which is associated with the inhibition of mitochondrial fission [12]. These studies indicate that OPA1 is an excellent therapeutic target for IRI; however, the challenge is that there is no effective agonist or promoter for OPA1 due to its complex protein molecular structure, and it is also very difficult to design small molecule drugs for it directly.

To address the issues above, we noticed that sodium–glucose cotransporter 2 inhibitors (SGLT2i), such as empagliflozin used in this study, have the potential to both reduce inflammation and promote OPA1 expression in renal IRI. As a novel class of hypoglycemic agents, SGLT2i have currently been available in a variety of marketed clinical agents, such as dapagliflozin, empagliflozin, and canagliflozin, and have been widely clinically with reliable clinical safety and application prospects [17, 18].

Xu et al. demonstrated that dapagliflozin prevented inflammation and improved autophagy in cultured human proximal renal tubular epithelial cells. Cells exposed to high glucose were characterized by the upregulation of several proinflammatory cytokines, including interleukin 1β (IL-1β), interleukin 6 (IL-6), and tumor necrosis factor alpha (TNF-α). Treatment with dapagliflozin significantly reduced the expression of all these cytokines in the cells and their concentrations in the culture supernatant [19]. In an animal model of type 2 diabetes, renal expression of PTX3 was significantly reduced with dapagliflozin treatments, thereby attenuating the inflammatory activity of macrophages, promoting the M2 phenotype of these cells, and downregulating nuclear factor κB (NF-κB), IL-1β, TNF-α, and monocyte chemoattractant protein 1 (MCP1) [20]. Based on previous studies, the hypothesis that empagliflozin can attenuate the inflammatory process triggered by renal IRI is reasonable.

In terms of the potential impact of SGLT2i on OPA1, a study in the laboratory of Mark E. Cooper, Australia, based on mice model of diabetes, found that SGLT2i maintained homeostasis of mitochondrial structure and function by increasing the expression of mitochondrial fusion factors OPA1 and Mitochondrial fusion factor 2 (Mfn2), and inhibiting the expression of mitochondrial fission protein (Fis1) in mouse cardiomyocytes [21]. Honda et al. from Yokohama University in Japan found that SGLT2i promoted OPA1 expression in obese mouse hepatocytes and improved hepatocyte injury and apoptosis through experiments in vivo and in vitro [22, 23]. These findings suggested that empagliflozin could promote OPA1 expression to regulate mitochondrial morphological dynamics, indicating that it might present desired therapeutic effects in IRI. SGLT2 is highly expressed in renal tubular cells, which is the main site of action of empagliflozin [24]. Thus, the diabetic drug empagliflozin may exhibit better selectivity and better IRI control in the kidney compared with the heart and liver.

However, the specific molecular biological mechanism by which SGLT2i regulates OPA1 expression remains unknown. It has been shown that the adenosine monophosphate-activated kinase (AMP-activated kinase, AMPK) signaling pathway is a key pathway that regulates mitochondrial dynamics processes [25, 26]. Activation of the AMPK signaling pathway can affect mitochondrial fusion and division by regulating the expression of OPA1 and dynamin-related protein 1 (Drp1) [27]. A recent study at the University of Wyoming suggested that empagliflozin activates intracellular AMPK pathways, promotes OPA1 expression, and inhibits mitochondrial fission, thereby protecting against microvascular injury in the heart in diabetes [28]. In addition, Zhang Ying et al. found that melatonin could promote mitochondrial fusion through the AMPK–OPA1 pathway in a mouse model of myocardial ischemia–reperfusion injury, thus protecting the myocardium from IRI [29]. Moreover, it has been previously reported that SGLT2i activated AMPK in liver and vessel cells [30, 31]. A study on diabetic nephropathy also observed AMPK activation by canagliflozin [32]. Although these studies did not systematically elucidate the molecular mechanisms, they implied that SGLT2i may act through the AMPK–OPA1 pathway to influence the regulation of mitochondrial dynamics.

Thus, in our experiments, renal IRI mice and H/R cell model were conducted to explore the renal protective function of empagliflozin through inflammation reduction and the AMPK–OPA1 pathway.

## Methods

### Mouse disease model and experiment design

Male C57BL/6 mice were selected at 8–10 weeks of age. Mice were administered empagliflozin (PubChem CID: 11949646, dissolved in water) at a dosage of 30 mg/kg/day for 1 week before surgery. Instruments and operating table were sterilized 1 day before surgery. The mice were anesthetized intraperitoneally with 10% chloral hydrate at 10 ml/100 g as the anesthetic agent. To avoid important organs, the injection was conducted into the lower abdomen and the needle was inserted at an angle of 30° to the abdominal cavity for intraperitoneal injection. After successful anesthesia, the mice were tied to the limbs and fixed on the operating table. A surgical incision of about 2 cm in length was made along the midline of the abdomen, the skin was incised, and the peritoneum was cut open to enter the abdominal cavity after freeing the skin layer. The intestine was gently pushed into the other side of the abdominal cavity with toothless forceps to find the kidney. The kidney pedicle was fully freed. The renal blood flow was blocked by clamping the kidney pedicle with a miniature arterial clip to prepare renal IRI model. The success criteria for the induction of IRI model was that kidney rapidly changed from bright red to purple–black. During the construction of the renal ischemia model, the mice were placed on a warm plate at 37 °C and the abdominal organs were kept moist by covering the abdominal incision with saline-soaked wet gauze. Heart rate, heart rhythm, respiration, and eye color were observed to understand the surgical condition of the mice. After 24 min of renal ischemia, the miniature arterial clamps were removed to restore blood flow to the kidneys. The kidneys were obtained at different time points according to the experimental needs and were later used for immunohistochemistry and RNA extraction.

### Mitochondrial isolation in renal tubular epithelial cells

Following the instructions of the Mitochondrial Isolation Kit (Thermo Scientific), primary renal tubular epithelial cells in the isolated culture dish were washed once with PBS, ten volumes of precooled PBS were added and chilled for 3 min, and all operations were performed on ice.

### Cellular hypoxia–reoxygenation model and experiment design

Human renal tubular epithelial cell lines (HK-2) (Guangzhou PythonBio Co., Ltd.) were used in cell rescue experiments. A hypoxic reoxygenation model was used in vitro to simulate renal tubular epithelial cell injury under reperfusion injury conditions. The hypoxic conditions were performed using fresh Hanks solution with 95% N_2_ and 5% CO_2_, while the pH of the solution was adjusted to approximately 6.8 by adding lactic acid to simulate the acidic environment under reperfusion injury. In this study, the hypoxic environment was maintained for 45 min, after which the cell culture dishes were placed under normoxic condition. The medium was changed to high-sugar Dulbecco’s modified Eagle medium [DMEM; containing 1% fetal calf serum (FBS)] to establish a reoxygenation model. In this study, the reoxygenation environment was maintained for 6 h. By establishing a hypoxic reoxygenation model, reperfusion injury of the microcirculation was simulated in vitro.

### Detection of apoptosis by flow cytometry

Annexin V was used in combination with propidium iodide to detect apoptosis. For cell surface staining, single-cell suspensions of mouse renal tubular epithelium treated with different concentrations of empagliflozin were incubated with the antibody mixture for 20 min at 4 °C. For intracellular staining, renal tubular cells were fixed in 4% paraformaldehyde at 4 °C for 20 min and subsequently permeabilized in Perm buffer overnight. The obtained data were analyzed using FACSDiva software (BD Biosciences).

### Hematoxylin–eosin staining and injury assessment

After deparaffinization, mouse renal tubular cells were presented via hematoxylin–eosin (HE) staining and sections were referred to experienced pathologists for blinded assessment of the following indicators: renal tubular vacuolization, peritubular or proximal tubule leukocyte infiltration, and tubule simplification. Injury cores were as follows: 0, normal; 1, mild; 2, moderate; 3, severe. A minimum of six fields were evaluated for each section [33].

### Detection of inflammatory factors by ELISA

Kidney cells from mice were divided into four groups: sham group, IRI group, sham group treated with empagliflozin, and IRI group treated with empagliflozin. The supernatant of the culture medium was collected after 48 h of incubation in the medium again. The levels of pro- and antiinflammatory cytokines in the culture medium were measured using enzyme linked immunosorbent assay (ELISA) kits according to the manufacturer’s instructions (R&D, Minneapolis, Minnesota, USA). The concentration of cytokines in the culture medium was calculated by measuring and correcting the optical density of the samples by subtracting the 570 nm reading from the 450 nm reading using an Emax Plus enzyme marker (Molecular Devices, San Jose, CA, USA).

### Indirect immunofluorescence detection

The protein expression in the cells was detected by indirect immunofluorescence. Cells were collected by cell crawl culture, fixed with 4% paraformaldehyde, and incubated with 10% normal goat serum for 30 min at room temperature to close the nonspecific binding site. Specific antibody was added and reserved overnight at 4 °C. Fluorescently labeled secondary antibody was added and incubated for 1 h at room temperature. Then 4,6-diamidino-2-phenylindole (DAPI) was added to stain cell nuclei for observation under a fluorescence microscope.

### TUNEL-staining in situ detection of apoptosis

Paraffin-embedded tissue sections were dewaxed twice for 5 min each by using xylene. Ethanol hydration was performed using graded alcohol (100%, 95%, 90%, 80%, 70%). Proteinase K (2%) was added to the sample. After reacting for 30 min at 21–37 °C, samples were rinsed twice in PBS and closed for 10 min in the closure solution (3% H_2_O_2_ dissolved in methanol). Then 50 µl TdT Enzyme Reaction Solution (1 µl Biotin-11-dUTP + 4 µl TdT Enzyme + 45 µl LEquilibration Buffer) was added dropwise to each sample for 60 min at 37 °C, protected from light and moistened. Next, 50 µl Streptavidin–horseradish peroxidase (HRP) working solution (0.5 µl Streptavidin–HRP + 99.5 µl PBS) and 100 µl 3,3′-diaminobenzidine (DAB) working solution (94 µl PBS + 5 µl DAB + 1 µl 30% H_2_O_2_) was added dropwise to each sample. Color development reaction was conducted at room temperature for 10 min. After rinsing three times with PBS, the samples were observed by a light microscope.

### Confocal microscope observation of mitochondrial morphology

Cell samples were perfused with 4% paraformaldehyde, fixed for 2–4 h or overnight at 4 °C. After gradient alcohol dehydration and xylene transparency, samples were blocked with 1% bovine serum albumin (BSA) for 1 h. Primary antibody was added and incubated for 1–2 h at 37 °C. Biotin-labeled secondary antibody was added dropwise and incubated for 40 min at 37 °C. Streptavidin–peroxidase was added and incubated for 40 min at room temperature. Samples were developed and photographed by a laser confocal microscope. ImageJ was used to analyze mitochondrial morphology.

### Detection of protein expression and analysis

Real-time PCR, RNA-sequencing, and western blot were used to detect protein expression levels. Immunofluorescence and laser confocal techniques were used to observe the subcellular localization of fusion proteins and the expression of each pathway protein. Differentially expressed genes (DEGs) identified from RNA-sequencing data were analyzed using an R package [34] and were defined as fold change > 2 and *Q*-value < 0.001. Kyoto Encyclopedia of Genes and Genomes (KEGG) pathway enrichment was analyzed using DAVID (https://david.ncifcrf.gov). Pathview was used to visualize KEGG [35, 36]. The expression of typical mitochondrial fusion and fission proteins was observed between groups. The cells were lysed with cytoplasmic and cytosolic lysates. The cytosolic and cytoplasmic proteins were extracted separately. The proteins in the nucleus and cytoplasm were detected separately by western blot. The detailed scheme of western blot can be found in our previous study [12].

### Statistical analysis

Data analysis was conducted by using statistical software, GraphPad Prism software (v8.0; La Jolla, CA, USA). Multiple group comparisons were conducted with a one-way ANOVA followed by Dunnett’s post hoc test or Tukey’s test. Differences with a *P*-value less than 0.05 were considered to be statistically significant and marked as *P < 0.05, **P < 0.01, and ***P < 0.001.

## Results

### Empagliflozin pretreatment prevented kidney injury triggered by renal IRI

We first established a renal IRI mice model to investigate the pathological mechanism of kidney injury caused by IRI. We drew blood from the eye canthus of mice to evaluate levels of serum creatinine (Scr) and blood urea nitrogen (BUN). Both the Scr and BUN levels were significantly elevated in IRI mice compared with control (Additional file 1: Fig. S1a), suggesting that renal IRI induced the elevation of Scr and BUN levels in mice. Renal pathological changes were subsequently detected via HE staining. Control mice had clear tubular structure, no obvious lesions, and low tubular interstitial injury scores, while IRI mice had unclear tubular morphology, incomplete structure, and elevated tubular interstitial injury scores (Additional file 1: Fig. S1b). These results suggest that ischemia–reperfusion caused renal pathological injury.

We then detected apoptosis in renal tubular epithelial cells. The apoptotic fraction in renal cells in IRI mice was significantly higher than in controls (Additional file 1: Fig. S1c), which suggests that IRI increased apoptosis in renal tissue. In addition, we detected the expression of relative genes for apoptosis and mitochondrial dynamics via qRT–PCR. Expression of *caspase3*, *caspase9*, and *Bax* was elevated while expression of *Bcl-2*, *Mfn1*, and *OPA1* decreased in IRI mice compared with control (Additional file 1: Fig. S1d). These results indicate that renal IRI upregulated genes related to apoptosis and mitochondrial fission and downregulated mitochondrial fusion-related genes in renal tissue.

Combined with the results above, we concluded that in renal IRI mice, levels of Scr and BUN, tubular interstitial injury, and apoptotic fraction in renal tissue were all elevated; the expression of *caspase3*, *caspase9*, and *Bax* in renal tissue increased; while the expression of *Bcl-2*, *Mfn1*, and *OPA1* decreased, which demonstrates that the IRI animal model was successfully established, and also indicated the potential relationship among pathological mechanisms of kidney injury, apoptosis, and mitochondrial fusion in renal tissue.

We then pretreated mice with empagliflozin to explore and verify the protection against IRI. Levels of Scr and BUN in control mice, IRI mice, and empagliflozin-treated IRI mice were evaluated. Scr and BUN levels were obviously elevated in IRI mice compared with controls. Interestingly, the levels decreased significantly in empagliflozin-treated IRI mice (Additional file 1: Fig. S2a), suggesting that empagliflozin pretreatment inhibited the elevation of Scr and BUN caused by renal IRI.

**Fig. 1:**
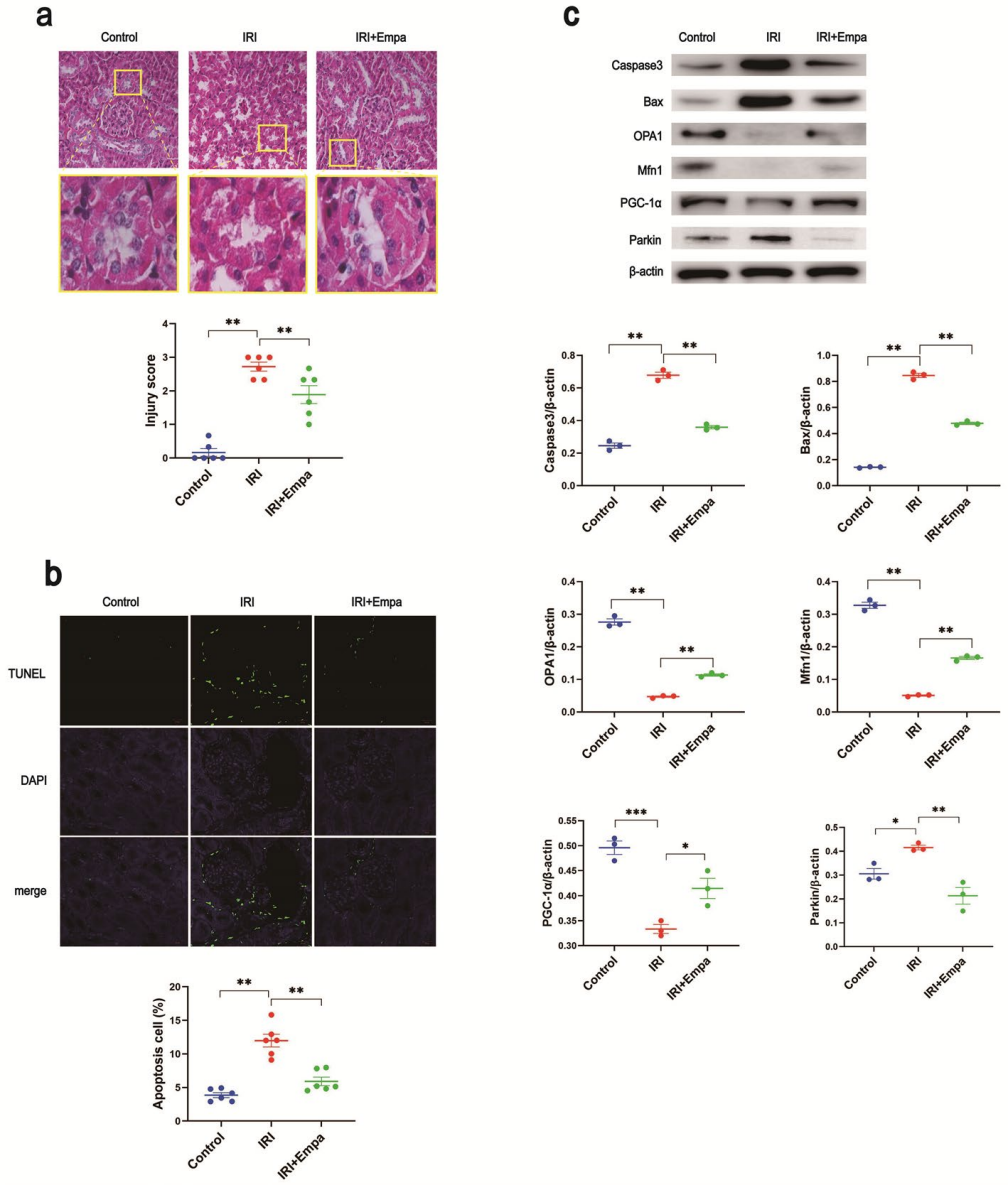
Empagliflozin pretreatment protected IRI mice from kidney injury. Empagliflozin was injected through the caudal vein in advance in empagliflozin-treated mice. **a** The renal pathological changes were presented by HE staining. Then, renal cell injury was quantified as injury scores. **b** TUNEL fluorescence assay was used to detect apoptosis and the strength of fluorescent signals presented apoptosis intensity. DAPI was used to label the nuclei. The colocalization of TUNEL and nuclei showed their positional relationships. **c** We detected the expression of caspase3, Bax, OPA1, and Mfn1 via western blot and used β-actin as the internal reference. The experiments were repeated independently at least three times, and the data were presented as the means ± SEM. Statistical significance with respect to the control was marked with *P < 0.05, **P < 0.01, or ***P < 0.001

As for pathological changes, the control mice had clear tubular structure, no obvious lesions, and low tubular interstitial injury scores. IRI mice had unclear tubular morphology, incomplete structure, and obviously elevated tubular interstitial injury scores compared with controls. Surprisingly, empagliflozin pretreated IRI mice had a significantly elevated number of clear tubular structures and much lower tubular interstitial injury scores than IRI mice (Fig. 1a). These results indicate that empagliflozin pretreatment inhibited tubular interstitial injury caused by renal IRI.

The apoptotic fraction in renal cells in IRI mice was significantly higher than in controls, while empagliflozin pretreatment noticeably reduced the apoptotic fraction in IRI mice (Fig. 1b). These results indicate that empagliflozin pretreatment inhibited the apoptosis in renal cells caused by IRI.

Compared with controls, the expression of *caspase3*, *caspase9*, and *Bax* was elevated, while the expression of *Bcl-2*, *Mfn1*, and *OPA1* decreased obviously in IRI mice. In accordance with expectation, the empagliflozin pretreated IRI mice had an apparent decrease in expression of *caspase3*, *caspase9*, and *Bax*, and a significant elevation in the expression of *Bcl-2*, *Mfn1*, and *OPA1* (Additional file 1: Fig. S2b), indicating that empagliflozin pretreatment reduced apoptosis and mitochondrial division in renal cells, promoting mitochondrial fusion.

Furthermore, gene expression, detected via western blot, showed that compared with controls, the expression of caspase3, Parkin, and Bax was elevated, while the expression of PGC-1α, Mfn1, and OPA1 decreased in IRI mice. As expected, empagliflozin pretreated IRI mice had decreased expression of caspase3, Parkin and, Bax and elevated expression of PGC-1α, Mfn1, and OPA1 (Fig. 1c). Likewise, the results indicated that empagliflozin pretreatment alleviated apoptosis and mitochondrial division in renal tissue.

From the above results, we concluded that we had successfully established the model and empagliflozin pretreatment could inhibit the downregulation of caspase3, caspase9, and Bax and the upregulation of Bcl-2, Mfn1, and OPA1 in IRI mice, as well as reduce tubular interstitial injury and apoptosis caused by renal IRI, thus alleviating kidney injury.

### IRI-triggered inflammation and mitochondrial division tendency was reduced by empagliflozin pretreatment at the gene expression level

To assess the intensity of the renal inflammatory response to empagliflozin during IRI, we examined the expression of proinflammatory/antiinflammatory factors, including proinflammatory factors IL-6, CCL-2, TNF-α, IFN-γ, and antiinflammatory factor IL-10, in renal tissues. The expression of IL-6, CCL-2, and TNF-α was significantly decreased in the IRI + empagliflozin group compared with the IRI + PBS group, while the expression of the antiinflammatory factor IL-10 was significantly increased (Additional file 1: Fig. S4). To further investigate the antiinflammatory mechanism of empagliflozin in renal IRI, we examined and analyzed transcripts from the IRI group and empagliflozin-treated group and performed KEGG pathway enrichment analysis. From the bioinformatics analysis, combined with the effects observed in vitro and in vivo, we identified numerous immune-related signaling pathways as potential pathways for empagliflozin treatment (Additional file 1: Fig. S3a, b). We visualized the two pathways we were interested in in the KEGG pathway view (Additional file 1: Fig. S3c, d). Elevated expression of adhesion molecules is key to the aggregation of damaging immune cells by IRI, of which T cells are the main adaptive immune cells involved [3]. Visualization results showed that the expression of surface adhesion molecules decreased in empagliflozin-treated renal tubular cells compared with those in IRI conditions, as well as the expression of major histocompatibility complex (MHC) I and MHC II molecules that activate T-cell function (Additional file 1: Fig. S3c, d).

**Fig. 2:**
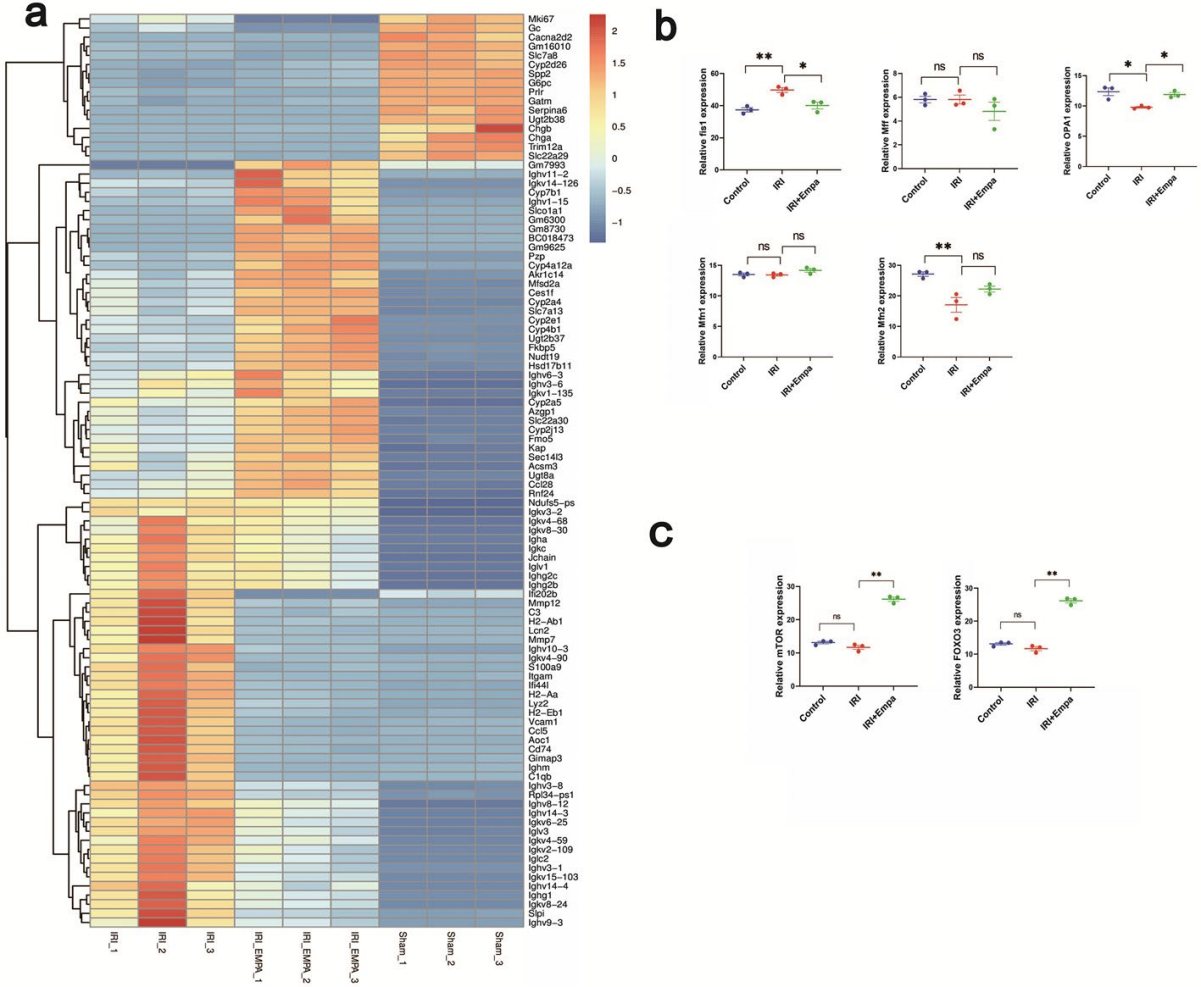
RNA sequencing of mitochondrial dynamics-related and AMPK-related genes. **a** Gene expression profiles in IRI model mice, empagliflozin-treated IRI mice, and sham mice were obtained by RNA sequencing. The results were visualized as a heat map of gene expression. **b** The relative expression of *Fis1*, *Mff*, *OPA1*, *Mfn1*, and *Mfn2*. **c** The relative expression of AMPK-related *mTOR* and *FOXO3*. The experiments were repeated independently at least three times, and the data were presented as the means ± SEM. Statistical significance with respect to control was marked with *P < 0.05, **P < 0.01 or ***P < 0.001

In gene expression profiles, there were total 2027 upregulated genes and 536 downregulated genes in IRI mice compared with control, and 261 upregulated genes and 536 downregulated genes were observed in empagliflozin-treated IRI mice compared with IRI model mice (Fig. 2a).

We then focused on the relative expression of *Fis1*, *Mff*, *OPA1*, *Mfn1*, and *Mfn2* among the sequencing results. Compared with controls, in IRI model mice, *Fis1* expression was upregulated, *OPA1* and *Mfn2* expression was downregulated, and there was no significant change observed in the expression of *Mff* and *Mfn1*. Compared with the IRI model mice, in empagliflozin-treated mice, *Fis1* expression was downregulated, *OPA1* expression was upregulated, while there was no significant change in the expression of the other genes (Fig. 2b). These results show that IRI treatment downregulated *OPA1* expression and upregulated *Fis1* expression in renal tissue, thus inhibiting mitochondrial fusion and promoting division, while empagliflozin pretreatment reversed the condition.

The sequencing results also showed the relative expression of *mTOR* and *FOXO3*, which were AMPK related and positively correlated with AMPK pathway activation. In IRI mice, there were no changes in their relative expression compared with control; however, empagliflozin pretreatment obviously elevated the expression of *mTOR* and *FOXO3* (Fig. 2c). These results suggest that although IRI had no effect on AMPK pathway activity, empagliflozin activated it.

RNA sequencing showed significant changes in inflammatory and mitochondrial dynamics-related factors after empagliflozin pretreatment. In particular for the latter, the AMPK pathway and factors such as *OPA1*, which corroborates previous studies and guided us to further carry out exploration of the mitochondrial dynamics in this work.

### Empagliflozin effectively inhibited the shortening of mitochondria in HK-2 cells under H/R

We initially determined the ideal empagliflozin dosing schedule. Using flow cytometry and the cell counting kit-8 (CCK8) assay, we optimized the processing time and concentration of empagliflozin for H/R cellular experiments. Four experimental groups and a control were set up to treat HK-2 cells: H/R, H/R + empagliflozin (50 nM), H/R + empagliflozin (100 nM), and H/R + empagliflozin (500 nM). The results showed that empagliflozin (100 nM) and empagliflozin (500 nM) protected HK-2 cells from H/R-induced apoptosis noticeably, while the protective effect of empagliflozin (50 nM) was comparably modest (Additional file 1: Fig. S5). As 500 nM Empagliflozin treatment for 72 h reduced the apoptotic fraction to the largest extent, this was chosen as the dosing schedule in our subsequent cellular experiments.

**Fig. 3:**
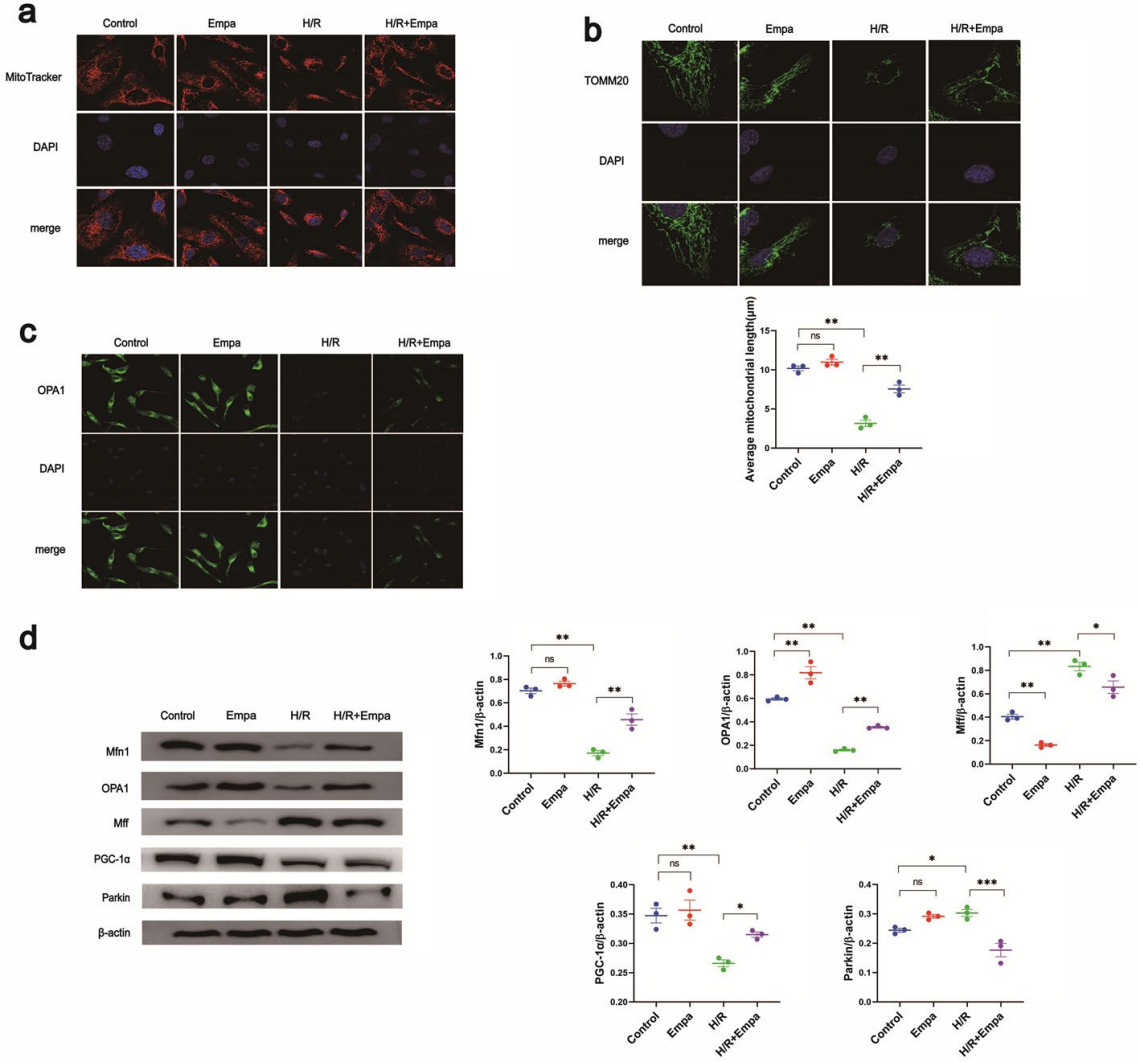
Empagliflozin effectively inhibited the shortening of mitochondria and promoted the expression of OPA1 in HK-2 cells under H/R. **a** Mito-Tracker Red CMXRos was used to mark mitochondria with bioactivity specifically. DAPI was used to label the nuclei. The colocalization of mitochondria and nuclei showed their positional relationships. **b** TOMM20 pAb was used to detect levels of TOMM20 protein. DAPI was used to label the nuclei. The colocalization of mitochondria and nuclei showed their positional relationships. **c** We detected the expression of OPA1 via immunofluorescence. **d** The expression of Mfn1, OPA1, and Mff was then detected via western blot, using β-actin as the internal reference. The results were subsequently quantified. The experiments were repeated independently at least three times, and the data were presented as the means ± SEM. Statistical significance with respect to control was marked with *P < 0.05, **P < 0.01 or ***P < 0.001

Firstly, mitochondrial morphology was investigated. Mitochondria were high in number and evenly distributed in control cells. After treating them with empagliflozin for 72 h, no significant changes were observed in mitochondrial number, distribution, or morphology. In the H/R condition, mitochondria in HK-2 cells were markedly reduced in number, shortened in length, and clustered. However, with empagliflozin treatment for 72 h, mitochondria in H/R treated cells were clearly elevated in number and length and evenly distributed (Fig. 3a).

We then investigated mitochondrial changes in terms of length exclusively via immunofluorescence. There were no significant changes in mitochondrial length after empagliflozin treatment compared with control. In the H/R condition, cells showed dotted or short rod-like mitochondrial staining and mitochondria were short in length. When treated with empagliflozin, mitochondria were elevated in length (Fig. 3b). The above results indicate that empagliflozin could effectively inhibit the shortening of mitochondria under H/R.

### OPA1 expression was elevated by empagliflozin in HK-2 cells under H/R

The expression of OPA1 was evaluated. Empagliflozin treatment had no significant effect on OPA1 expression compared with controls when normally cultured, whereas OPA1 expression decreased under H/R, and empagliflozin treatment partially relieved the condition (Fig. 3c). These results suggest that Empagliflozin could promote OPA1 expression in IRI renal tubular epithelial cells.

The expression of Mfn1, OPA1, Mff, PGC-1α, and Parkin was detected via western blot, using β-actin as the internal reference, and the results were subsequently quantified. The expression of Mff decreased significantly in empagliflozin-treated mice when normally cultured compared with control. In the H/R condition, Mff expression was elevated, while empagliflozin slightly inhibited the elevation. The expression of Mfn1, PGC-1α, and Parkin showed no significant difference between control and empagliflozin-treated mice, while in the H/R condition, Mfn1 and PGC-1α expression sharply decreased and empagliflozin treatment significantly elevated the expression, whereas for Parkin, the exact opposite occurred. The expression of OPA1 in empagliflozin mice was significantly higher than in controls. H/R treatment lowered the OPA1 expression compared with the control and empagliflozin treatment it (Fig. 3d).

### Empagliflozin protected mitochondria by promoting OPA1 in IRI-like conditions induced by OPA1 knockdown in HK-2 cells

**Fig. 4:**
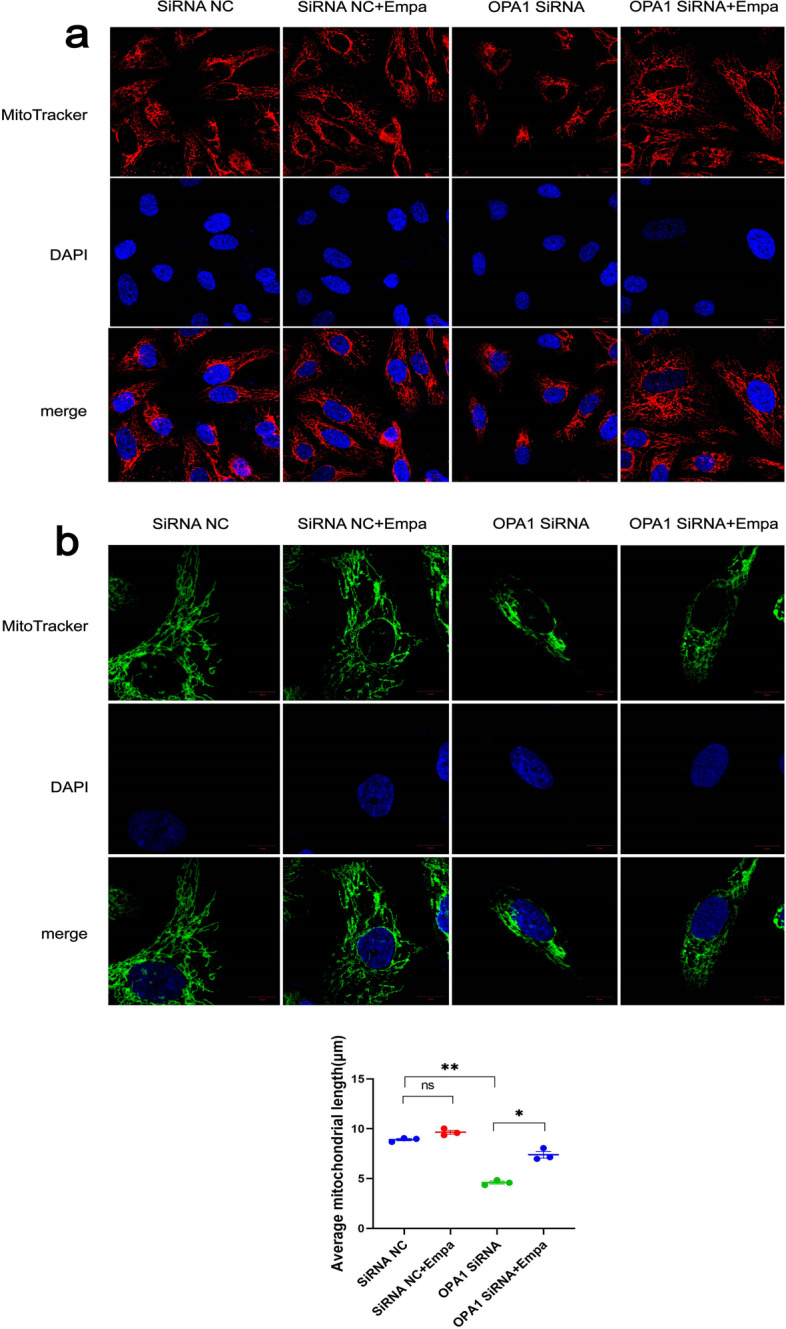
Empagliflozin positively protected mitochondria in an OPA1-knockdown, IRI-like condition in HK-2 cells. **a** Mito-Tracker Red CMXRos was specifically used to mark mitochondria with bioactivity. DAPI was used to label the nuclei. The colocalization of mitochondria and nuclei showed their positional relationships. Mitochondrial morphology was then quantified. **b** The mitochondrial length was detected via immunofluorescence. TOMM20 pAb was used to detect levels of TOMM20 protein. DAPI was used to label the nuclei. The colocalization of mitochondria and nuclei showed their positional relationships. The experiments were repeated independently at least three times, and the data were presented as the means ± SEM. Statistical significance with respect to controls was marked with *P < 0.05, **P < 0.01, or ***P < 0.001

We observed the morphology of mitochondria under different treatments. In the control, mitochondria were high in number and evenly distributed. With empagliflozin treatment for 72 h, no significant changes were observed in mitochondrial number, distribution, or morphology compared with controls. After OPA1 knockdown via OPA1 small interfering (si)RNA treatment, mitochondria in HK-2 cells were reduced in number, shortened in length, and clustered, consistent with the mitochondria in IRI. Subsequent empagliflozin treatment reversed the mitochondria injury condition caused by OPA1 knockdown (Fig. 4a). These results show that OPA1 knockdown caused an IRI-like condition in renal tissue, and that empagliflozin positively protected mitochondria in IRI-like conditions induced by OPA1 knockdown in renal tubular epithelial cells.

Similarly, we observed mitochondria in terms of length exclusively, as shown in Fig. 4b. There were no significant changes in mitochondrial length after empagliflozin treatment compared with control. Mitochondria in OPA1 siRNA-treated cells showed dotted or short rod-like mitochondrial staining, indicating mitochondrial division. This injury phenotype in OPA1 siRNA-treated cells was partially relieved after adding empagliflozin (Fig. 4a). The above results indicate that knockdown of OPA1 expression could promote mitochondrial division, while empagliflozin could inhibit mitochondrial division, effectively alleviating the phenotype of mitochondrial IRI caused by OPA1 knockdown.

### AMPK inhibitor blocked the promotion of OPA1 by empagliflozin

**Fig. 5:**
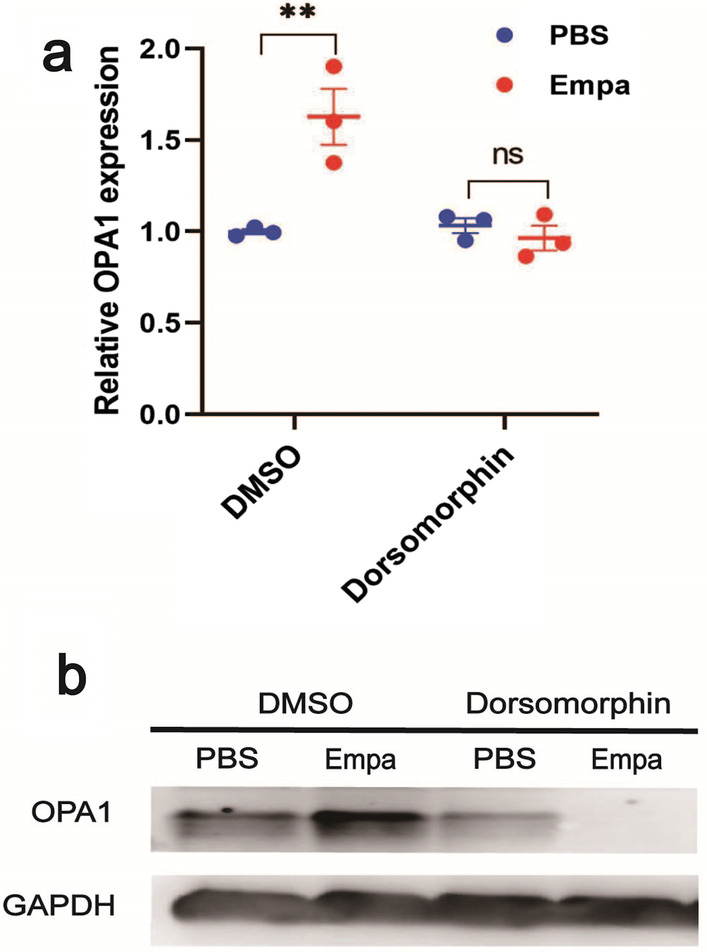
AMPK inhibitor blocked the promotion of OPA1 by empagliflozin. **a** H/R HK-2 cells were constructed, and supplemented with empagliflozin or PBS along with AMPK inhibitor Dorsomorphin or DMSO. After 24 h culture, RNA was extracted and *OPA1* expression was detected by quantitative PCR. **b** After the same treatment as **a**, total protein samples were extracted, and immunoblot western blot was performed to detect OPA1 levels. The experiments were repeated independently at least three times, and the data were presented as the means ± SEM. Statistical significance with respect to control was marked with *P < 0.05, **P < 0.01, or ***P < 0.001

To preliminarily determine the role of AMPK in empagliflozin protection against renal IRI, we constructed an HK-2 cells H/R model. H/R cells were then treated with AMPK inhibitor Dorsomorphin (Compound C) or control dimethyl sulfoxide (DMSO). Subsequently, protein and RNA samples were extracted, and the protein levels and mRNA levels of *OPA1* were detected via immunoblotting assay and quantitative PCR assay, respectively (Fig. 5a, b). Adding PBS as a control, DMSO-treated H/R cells reacted to empagliflozin, and had a significant elevation in OPA1 expression both in immunoblotting and quantitative PCR, while Dorsomorphin-treated cells showed no reaction to empagliflozin. These results demonstrate that the promotion of OPA1 expression by empagliflozin is dependent on the AMPK signaling pathway.

## Discussion

SGLT2i, like empagliflozin, have been shown to alleviate and protect against various kidney diseases in clinical studies [37, 38]. However, the mechanism has not been clearly elucidated so far. In our experiments, empagliflozin treatment reduced inflammatory effects, enhanced the mitochondrial fusion process, and affected mitochondrial homeostasis in renal tubular epithelial cells, ultimately reducing renal injury and tubular epithelial cell apoptosis during ischemia–reperfusion injury. We showed the following: (i) in IRI mice, mitochondria in renal tubular epithelial cells were damaged and empagliflozin pretreatment protected IRI mice from kidney injury; (ii) in the inflammatory signature assay, empagliflozin pretreatment caused a significant decrease in proinflammatory factors and a significant increase in antiinflammatory factors; (iii) in H/R cellular experiments, empagliflozin treatment increased the expression of OPA1, which plays a key role in mitochondrial fusion; (iv) in an IRI-like condition induced by OPA1 knockdown, empagliflozin treatment inhibited the shortening of mitochondria and improved mitochondrial fusion in cultured renal tubular epithelial cells; (v) in AMPK inhibition experiments, the expression of OPA1 promoted by empagliflozin was blocked by Dorsomorphin. These results indicate the renoprotective and therapeutic effects of empagliflozin and its molecular mechanisms in renal IRI.

To understand the renoprotective and therapeutic effects of empagliflozin, we need to explore cellular and animal models separately, and consider the effects on the extracellular and intracellular environments. In in vivo experiments, through the IRI mice model, we demonstrated that empagliflozin pretreatment can reduce inflammation and reverse the mitochondrial division caused by renal IRI. In in vitro experiments, through rescue experiments and H/R modeling, we demonstrated that empagliflozin treatment can prevent and protect mitochondria shortening under H/R or in IRI-like conditions through promoting OPA1 expression, and through AMPK inhibition experiments, we demonstrated that the AMPK signaling pathway is involved with empagliflozin to promote OPA1 expression. These data confirm the critical function of empagliflozin for improving renal IRI and implied its possible signaling pathway.

Inflammation reduction and the AMPK–OPA1 pathway are the focus of our attention, and other studies provide support for our experiments. A large clinical trial conducted by Wanner et al. showed a significant reduction in the incidence of kidney disease, risk of progression, and risk of kidney replacement therapy in the SGLT2i treatment group [39]. Several experiments point to SGLT2i as an antioxidant and antiinflammatory agent in models of kidney injury [40]. Xu et al. demonstrated that Dapagliflozin activated AMPK and reduced the activity of mammalian targets of rapamycin (mTOR), thereby attenuating inflammatory effects and improving autophagy in human proximal renal tubular epithelial cells [19]. Birnbaum et al. detected that the renal expression levels of IL-1β, IL-6, and TNF-α were attenuated by Dapagliflozin in an animal model of diabetes-activated renal inflammation [41].

However, unlike inflammation, which has received as much attention, the effect of SGLT2i on mitochondrial and morphological dynamics within renal tubular cells is a question that deserves deeper investigation. Mitochondrial fusion and division have been shown to be mediated by a variety of kinetic-related GTPases, including Drp1, Fis1, Mfn1, and OPA1. Among them, OPA1 is the main fusion factor that regulates the mitochondrial fusion process [42–44]. In our previous study, we also found that OPA1 could improve renal IRI by maintaining mitochondrial homeostasis in renal tubular cells [12]. A previous study conducted by Takagi et al. had similar conclusions to ours. They confirmed that SGLT2i can improve mitochondrial morphology and restore mitochondrial biogenesis-related molecules in mice fed with a high-fat diet (HFD). High glucose induced by HFD can cause mitochondrial defects, with deficiencies in OPA1 and Mfn2. SGLT2i restore mitochondrial biogenesis in high glucose associated with the normalization of OPA1 and Mfn2 levels [45]. Also, as mentioned before, Honda et al. and the laboratory of Mark E. Cooper found that SGLT2i can promote OPA1 expression [21–23]. The work of Zhou et al. and Zhang et al. strongly suggested that AMPK may be the signaling pathway through which SGLT2i affect OPA1 expression. These previous studies provided the theoretical basis for our experiments and promising reliability for our conclusions.

However, there were certain limitations to our present study. We have demonstrated the inflammation-reducing effect of empagliflozin on renal IRI. However, we only stopped at a microarray analysis of cellular genes and bioinformatics analysis for the molecular mechanism of how empagliflozin attenuates inflammation in renal IRI. Therefore, more laboratory data are needed to elucidate the specific mechanisms. We have demonstrated the promotion of OPA1 expression by empagliflozin under IRI conditions to protect renal cells from injury and enhance mitochondrial fusion in OPA1-knockdown renal tubular epithelial cells, but more experiments may be required to further validate the dependence. The same problem exists in the signaling pathway. We have demonstrated that the AMPK–OPA1 pathway is involved in empagliflozin exerting a renal IRI protective mechanism. Further experiments are still needed to explore and confirm the complete signaling pathway of empagliflozin promoting OPA1 expression. In short, the complete mechanisms of renal IRI protective effects of empagliflozin need to be further verified and elucidated.

The limitations of this study will guide our future work. In addition to continuing to elucidate the molecular mechanisms by which Empagliflozin attenuates inflammation and its dependence on the AMPK–OPA1 signaling pathway, we suggest the following aspects be examined, further investigating the renal functions of empagliflozin: (i) whether Empagliflozin treatment affects posttranslational modifications of AMPK protein complexes and (ii) whether the degree of AMPK activation differs with different SGLT2i drugs [31]. IRI and mitochondrial dynamics are both complex processes and there may be other possible pathways involved in SGLT2i-mediated renal protection. Chang et al. and Ndibalema et al. suggested a possible role for SGLT2i in the regulation of hypoxia-inducible factor 1-α (HIF1α) in renal IRI protection, but the mechanism has not been clearly elaborated and demonstrated [33, 46]. We should keep an open attitude toward the investigation of signaling pathways, not limiting ourselves to one pathway or molecule, and any useful hints are worthy of our investigation. Meanwhile, empagliflozin, as a hypoglycemic agent, is mainly designed for SGLT2. Optimization and redesign of its structure may have unexpected effects on renal protection.

## Conclusions

We have confirmed the important function of empagliflozin in renal IRI damage. Empagliflozin treatment improves renal tubular epithelial cell necrosis under IRI conditions by reducing inflammation and improving mitochondrial morphology through promoting the AMPK–OPA1 pathway.

## Supplementary Information

**Additional file 1: Fig. S1.** Renal IRI triggered kidney injury in model mice. **Fig. S2.** Biochemical and genetic testing in empagliflozin pretreatment. **Fig. S3.** Bioinformatics analysis of the antiinflammatory response caused by empagliflozin. **Fig. S4.** Detection of inflammatory response in kidney tissue during IRI. **Fig. S5.** Analysis of the appropriate dose of empagliflozin by flow cytometry.

## Data Availability

Core data has been provided in main text. The data can be available on a reasonable request.

## Abbreviations

AMPK: Adenosine 5-monophosphate (AMP)-activated protein kinase
Bax: Bcl2-Associated X (Bcl-2, B-cell lymphoma-2)
BSA: Bovine serum albumin
BUN: Blood urea nitrogen
CCK8: Cell counting kit-8
DAPI: 4,6-Diamidino-2-phenylindole
DEGs: Differentially expressed genes
DGF: Delayed graft function
DMEM: Dulbecco’s modified Eagle medium
DMSO: Dimethyl sulfoxide
Drp1: Dynamin-related protein 1
FBS: Fetal calf serum
Fis1: Mitochondrial fission 1 protein
FOXO3: Forkhead box O3
HE: Hematoxylin–eosin
HFD: High-fat diet
HK-2: Human renal tubular epithelial cell line
HRP: Horseradish peroxidase
H/R: Hypoxia/reoxygenation
IRI: Ischemia–reperfusion injury
KEGG: Kyoto Encyclopedia of Genes and Genomes
Mff: Mitochondrial fission factor
Mfn1: Mitofusin 1
Mfn2: Mitofusin 2
Mito Tracker: Mito-Tracker Red CMXRos
mTOR: Mammalian target of rapamycin
NC: Negative control
OPA1: Optic atrophy protein 1
PBS: Phosphate buffered saline
PCR: Polymerase chain reaction
PGC-1α: Peroxisome proliIerator-activated receptor γ coactivator 1 alpha
PI: Propidium iodide
Scr: Serum creatinine
SGLT2i: Sodium–glucose cotransporter 2 inhibitor
TOMM20: Mitochondrial import receptor subunit TOM20 homolog
TUNEL: Terminal deoxynucleotidyl transferase (TdT)-mediated dUTP nick end labeling

## References

[1] Nieuwenhuijs-Moeke GJ, Pischke SE, Berger SP, Sanders JSF, Pol RA, Struys MMRF (2020). Ischemia and reperfusion injury in kidney transplantation: relevant mechanisms in injury and repair. JCM.

[2] Bahl D, Haddad Z, Datoo A, Qazi YA (2019). Delayed graft function in kidney transplantation. Curr Opin Organ Transplant.

[3] Jang HR, Rabb H (2015). Immune cells in experimental acute kidney injury. Nat Rev Nephrol.

[4] Jang HR, Rabb H (2009). The innate immune response in ischemic acute kidney injury. Clin Immunol.

[5] Jang HR, Ko GJ, Wasowska BA, Rabb H (2009). The interaction between ischemia–reperfusion and immune responses in the kidney. J Mol Med.

[6] Abukar Y, Ramchandra R, Hood SG, McKinley MJ, Booth LC, Yao ST (2018). Increased cardiac sympathetic nerve activity in ovine heart failure is reduced by lesion of the area postrema, but not lamina terminalis. Basic Res Cardiol.

[7] Bocci M, Sjölund J, Kurzejamska E, Lindgren D, Marzouka NAD, Bartoschek M (2019). Activin receptor-like kinase 1 is associated with immune cell infiltration and regulates CLEC14A transcription in cancer. Angiogenesis.

[8] Vásquez-Trincado C, García-Carvajal I, Pennanen C, Parra V, Hill JA, Rothermel BA (2016). Mitochondrial dynamics, mitophagy and cardiovascular disease: mitochondria and cardiovascular disease. J Physiol.

[9] Ong SB, Hausenloy DJ (2010). Mitochondrial morphology and cardiovascular disease. Cardiovasc Res.

[10] Chrifi I, Louzao-Martinez L, Brandt MM, van Dijk CGM, Bürgisser PE, Zhu C (2019). CMTM4 regulates angiogenesis by promoting cell surface recycling of VE-cadherin to endothelial adherens junctions. Angiogenesis.

[11] Huang J, Xie P, Dong Y, An W (2021). Inhibition of Drp1 SUMOylation by ALR protects the liver from ischemia-reperfusion injury. Cell Death Differ.

[12] Wang Q, Xu J, Li X, Liu Z, Han Y, Xu X (2019). Sirt3 modulate renal ischemia-reperfusion injury through enhancing mitochondrial fusion and activating the ERK-OPA1 signaling pathway. J Cell Physiol.

[13] Coverstone ED, Bach RG, Chen L, Bierut LJ, Li AY, Lenzini PA (2018). A novel genetic marker of decreased inflammation and improved survival after acute myocardial infarction. Basic Res Cardiol.

[14] Darden J, Payne LB, Zhao H, Chappell JC (2019). Excess vascular endothelial growth factor-A disrupts pericyte recruitment during blood vessel formation. Angiogenesis.

[15] Lee Y, Jeong SY, Karbowski M, Smith CL, Youle RJ (2004). Roles of the mammalian mitochondrial fission and fusion mediators Fis1, Drp1, and Opa1 in apoptosis. Mol Biol Cell.

[16] Arnoult D, Grodet A, Lee YJ, Estaquier J, Blackstone C (2005). Release of OPA1 during apoptosis participates in the rapid and complete release of cytochrome c and subsequent mitochondrial fragmentation. J Biol Chem.

[17] Ni L, Yuan C, Chen G, Zhang C, Wu X (2020). SGLT2i: beyond the glucose-lowering effect. Cardiovasc Diabetol.

[18] Mima A (2022). Mitochondria-targeted drugs for diabetic kidney disease. Heliyon.

[19] Xu J, Kitada M, Ogura Y, Liu H, Koya D (2021). Dapagliflozin restores impaired autophagy and suppresses inflammation in high glucose-treated HK-2 cells. Cells.

[20] Elkazzaz SK, Khodeer DM, El Fayoumi HM, Moustafa YM (2021). Role of sodium glucose cotransporter type 2 inhibitors Dapagliflozin on diabetic nephropathy in rats; Inflammation, angiogenesis and apoptosis. Life Sci.

[21] Warren AM, Knudsen ST, Cooper ME (2019). Diabetic nephropathy: an insight into molecular mechanisms and emerging therapies. Expert Opin Ther Targets.

[22] Honda Y, Imajo K, Kato T, Kessoku T, Ogawa Y, Tomeno W (2016). The selective SGLT2 inhibitor ipragliflozin has a therapeutic effect on nonalcoholic steatohepatitis in mice. PLoS ONE.

[23] Jinnouchi H, Nozaki K, Watase H, Omiya H, Sakai S, Samukawa Y (2016). Impact of reduced renal function on the glucose-lowering effects of luseogliflozin, a selective SGLT2 inhibitor, assessed by continuous glucose monitoring in Japanese patients with type 2 diabetes mellitus. Adv Ther.

[24] Cowie MR, Fisher M (2020). SGLT2 inhibitors: mechanisms of cardiovascular benefit beyond glycaemic control. Nat Rev Cardiol.

[25] Toyama EQ, Herzig S, Courchet J, Lewis TL, Losón OC, Hellberg K (2016). AMP-activated protein kinase mediates mitochondrial fission in response to energy stress. Science.

[26] Zhang CS, Lin SC (2016). AMPK promotes autophagy by facilitating mitochondrial fission. Cell Metab.

[27] Li J, Wang Y, Wang Y, Wen X, Ma XN, Chen W (2015). Pharmacological activation of AMPK prevents Drp1-mediated mitochondrial fission and alleviates endoplasmic reticulum stress-associated endothelial dysfunction. J Mol Cell Cardiol.

[28] Zhou H, Wang S, Zhu P, Hu S, Chen Y, Ren J (2018). Empagliflozin rescues diabetic myocardial microvascular injury via AMPK-mediated inhibition of mitochondrial fission. Redox Biol.

[29] Zhang Y, Wang Y, Xu J, Tian F, Hu S, Chen Y (2019). Melatonin attenuates myocardial ischemia-reperfusion injury via improving mitochondrial fusion/mitophagy and activating the AMPK-OPA1 signaling pathways. J Pineal Res.

[30] Mancini SJ, Boyd D, Katwan OJ, Strembitska A, Almabrouk TA, Kennedy S (2018). Canagliflozin inhibits interleukin-1β-stimulated cytokine and chemokine secretion in vascular endothelial cells by AMP-activated protein kinase-dependent and -independent mechanisms. Sci Rep.

[31] Hawley SA, Ford RJ, Smith BK, Gowans GJ, Mancini SJ, Pitt RD (2016). The Na+/glucose cotransporter inhibitor canagliflozin activates AMPK by inhibiting mitochondrial function and increasing cellular AMP levels. Diabetes.

[32] Inoue MK, Matsunaga Y, Nakatsu Y, Yamamotoya T, Ueda K, Kushiyama A (2019). Possible involvement of normalized Pin1 expression level and AMPK activation in the molecular mechanisms underlying renal protective effects of SGLT2 inhibitors in mice. Diabetol Metab Syndr.

[33] Chang YK, Choi H, Jeong JY, Na KR, Lee KW, Lim BJ (2016). Dapagliflozin, SGLT2 inhibitor, attenuates renal ischemia-reperfusion injury. PLoS ONE.

[34] Wang L, Feng Z, Wang X, Wang X, Zhang X (2010). DEGseq: an R package for identifying differentially expressed genes from RNA-seq data. Bioinformatics.

[35] Luo W, Brouwer C (2013). Pathview: an R/Bioconductor package for pathway-based data integration and visualization. Bioinformatics.

[36] Luo W, Pant G, Bhavnasi YK, Blanchard SG, Brouwer C (2017). Pathview Web: user friendly pathway visualization and data integration. Nucleic Acids Res.

[37] Mima A (2018). Renal protection by sodium-glucose cotransporter 2 inhibitors and its underlying mechanisms in diabetic kidney disease. J Diabetes Complicat.

[38] Mima A (2021). Sodium-glucose cotransporter 2 inhibitors in patients with non-diabetic chronic kidney disease. Adv Ther.

[39] Wanner C, Inzucchi SE, Lachin JM, Fitchett D, von Eynatten M, Mattheus M (2016). Empagliflozin and progression of kidney disease in type 2 diabetes. N Engl J Med.

[40] Winiarska A, Knysak M, Nabrdalik K, Gumprecht J, Stompór T (2021). Inflammation and oxidative stress in diabetic kidney disease: the targets for SGLT2 inhibitors and GLP-1 receptor agonists. IJMS.

[41] Birnbaum Y, Bajaj M, Yang HC, Ye Y (2018). Combined SGLT2 and DPP4 inhibition reduces the activation of the Nlrp3/ASC inflammasome and attenuates the development of diabetic nephropathy in mice with type 2 diabetes. Cardiovasc Drugs Ther.

[42] Dohm JA, Lee SJ, Hardwick JM, Hill RB, Gittis AG (2003). Cytosolic domain of the human mitochondrial fission protein fis1 adopts a TPR fold. Proteins.

[43] Meeusen S, DeVay R, Block J, Cassidy-Stone A, Wayson S, McCaffery JM (2006). Mitochondrial inner-membrane fusion and crista maintenance requires the dynamin-related GTPase Mgm1. Cell.

[44] Meeusen S, McCaffery JM, Nunnari J (2004). Mitochondrial fusion intermediates revealed in vitro. Science.

[45] Takagi S, Li J, Takagaki Y, Kitada M, Nitta K, Takasu T (2018). Ipragliflozin improves mitochondrial abnormalities in renal tubules induced by a high-fat diet. J Diabetes Investig.

[46] Ndibalema A, Kabuye D, Wen S, Li L, Li X, Fan Q (2020). Empagliflozin protects against proximal renal tubular cell injury induced by high glucose via regulation of hypoxia-inducible factor 1-alpha. DMSO.

