# Supplementary material for: Empagliflozin improves renal ischemia–reperfusion injury by reducing inflammation and enhancing mitochondrial fusion through AMPK–OPA1 pathway promotion
Source: Cell Mol Biol Lett. 2023 May 18;28:42. doi: 10.1186/s11658-023-00457-6 (PMC10197452; doi:10.1186/s11658-023-00457-6)
Supplement: Supplementary file 1 — Additional file 1: Fig. S1. Renal IRI triggered kidney injury in model mice. Fig. S2. Biochemical and genetic testing in empagliflozin pretreatment. Fig. S3. Bioinformatics analysis of the antiinflammatory response caused by empagliflozin. Fig. S4. Detection of inflammatory response in kidney tissue during IRI. Fig. S5. Analysis of the appropriate dose of empagliflozin by flow cytometry. [file 11658_2023_457_MOESM1_ESM.docx]

**ADDITIONAL FILES**

**Empagliflozin improves renal ischemia‐reperfusion injury by reducing inflammation and enhancing mitochondrial fusion through AMPK-OPA1 pathway promotion**

Wenbo Yang^1*^, Xiaoli Li^2*^, Liujie He^3*^, Shuyang Zhu^3*^, Shicong Lai^1^, Xiao-peng Zhang^1^, Zixiong Huang^1^, Biyue Yu^4^, Chunping Cui^5^, Qiang Wang^1#^

^1^Department of Urology, Peking University People’s Hospital, Beijing 100044, China

^2^Department of the eighth healthcare, the Second Medical Center & National Clinical Research Center for Geriatric Diseases, Chinese PLA General Hospital. Beijing, 100853, P.R. China

^3^Naval Medical University, Shanghai 200433, China

^4^School of Life Sciences, Hebei University, Baoding, Hebei Province 071002, China

^5^State Key Laboratory of Proteomics, National Center for Protein Sciences (Beijing), Beijing Institute of Lifeomics, Beijing 100850, China

^*^These authors contributed equally to this article.

^#^Correspondence: Qiang Wang, MD, PhD, E-mail: [wq301135@126.com](mailto:wq301135@126.com)

**Supplementary Figures**

**
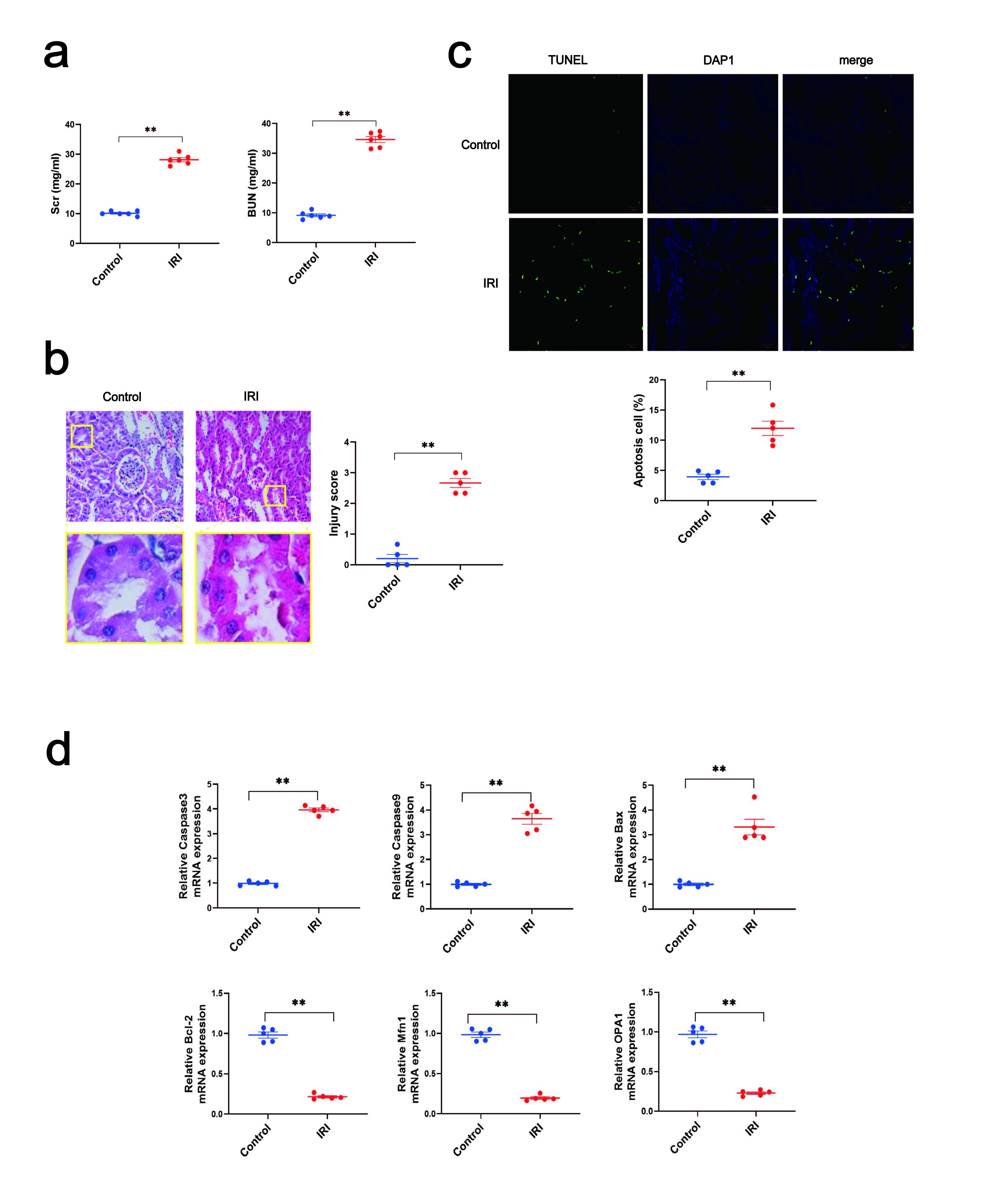
**

**Fig.S1 Renal IRI triggered kidney injury in model mice** (a) Scr and BUN levels were evaluated using ELISA. Blood was drawn from the eye canthus. (b) The renal pathological changes were presented by HE staining. Renal cell injury was then quantified as injury scores. (c) TUNEL [fluorescence assay](https://www.sciencedirect.com/topics/biochemistry-genetics-and-molecular-biology/fluorescence-assay) was used to detect apoptosis and the strength of fluorescent signals presented apoptosis intensity. DAPI was used to label the nuclei. The colocalization of TUNEL and nuclei showed their positional relationships. Then, TUNEL signals were quantified to obtain apoptotic fraction. (d) The expression of related genes was detected via qRT-PCR. The experiments were repeated independently at least three times, and the data were presented as the means±SEM. Statistical significance with respect to control was marked with *P < 0.05, **P < 0.01 or ***P < 0.001 respectively.

**
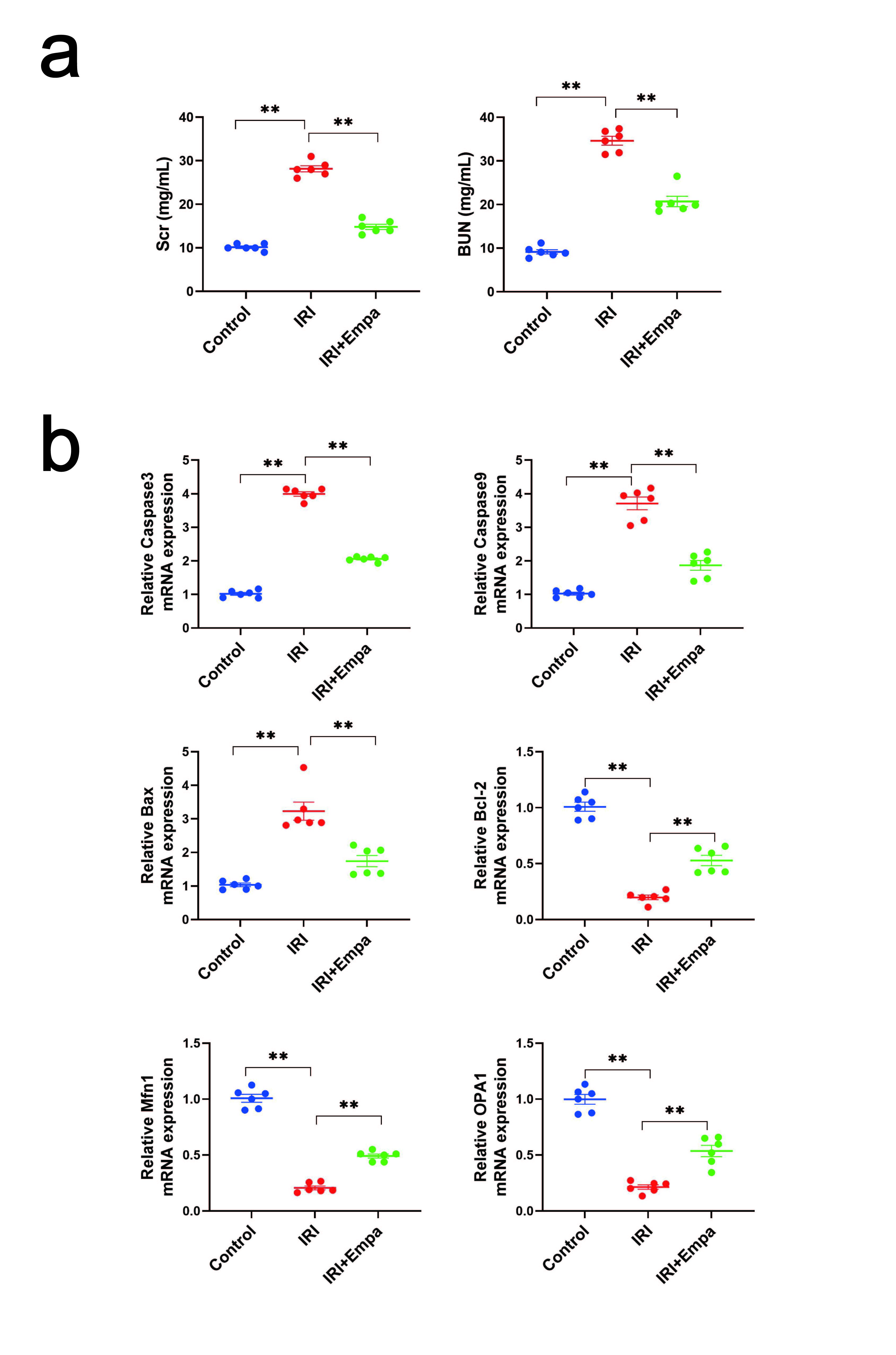
**

**Fig.S2 Biochemical and genetic testing in Empagliflozin pretreatment** (a) Levels of Scr and BUN were evaluated via ELISA. (b) The expression of genes was detected via qRT-PCR. The experiments were repeated independently at least three times, and the data were presented as the means±SEM. Statistical significance with respect to control was marked with *P < 0.05, **P < 0.01 or ***P < 0.001 respectively.

**
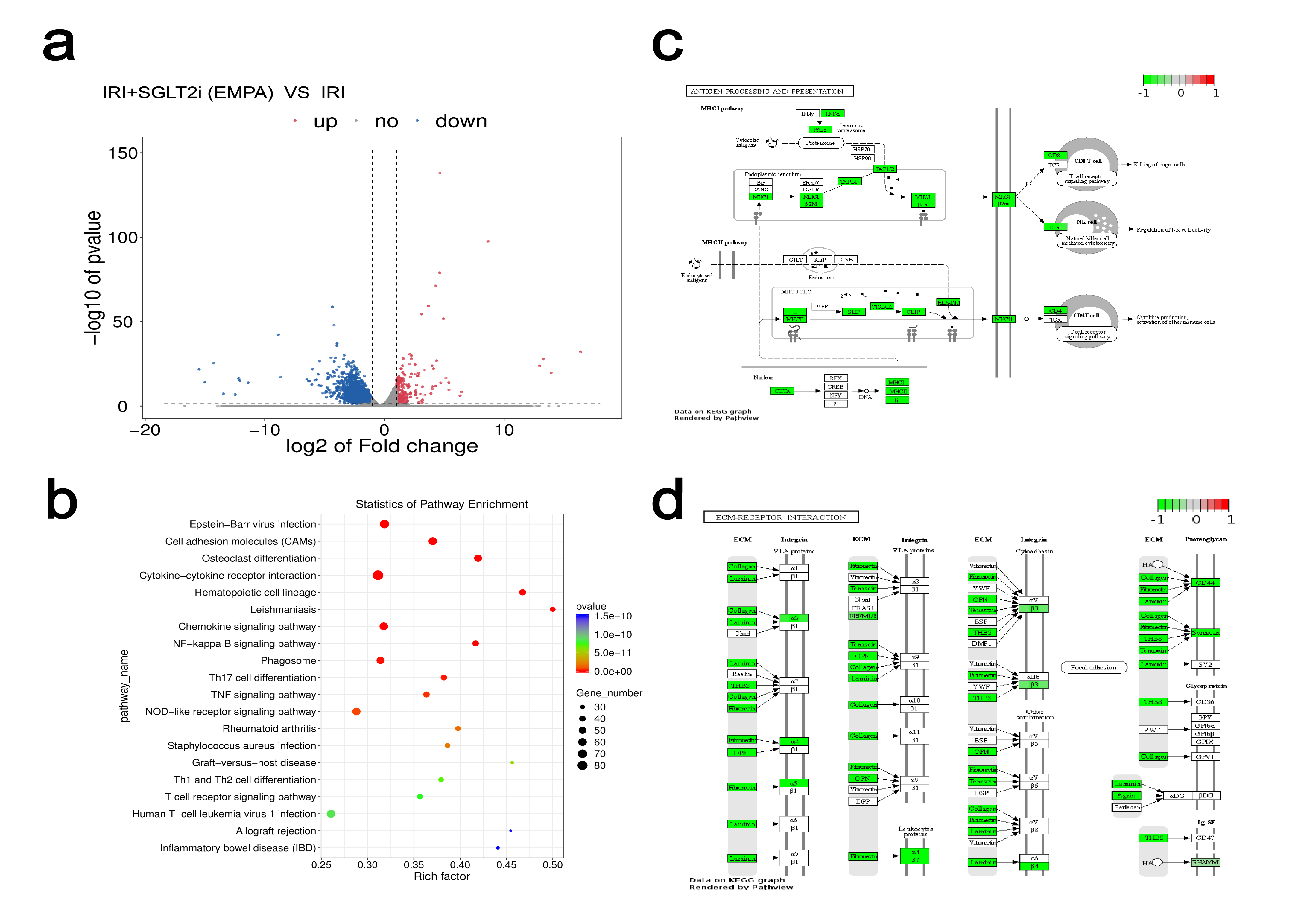
**

**Fig.S3 Bioinformatics analysis of the anti-inflammatory response caused by Empagliflozin** (a) Volcano plot of IRI-vs-pretreatment revealing up- and down-regulated DEGs after Empagliflozin pretreatment. (b) KEGG analysis of Empagliflozin pretreatment. (c) Visual view of the KEGG pathway for antigen processing and presentation. (d) Visual view of the KEGG pathway for ECM-receptor interaction.

**
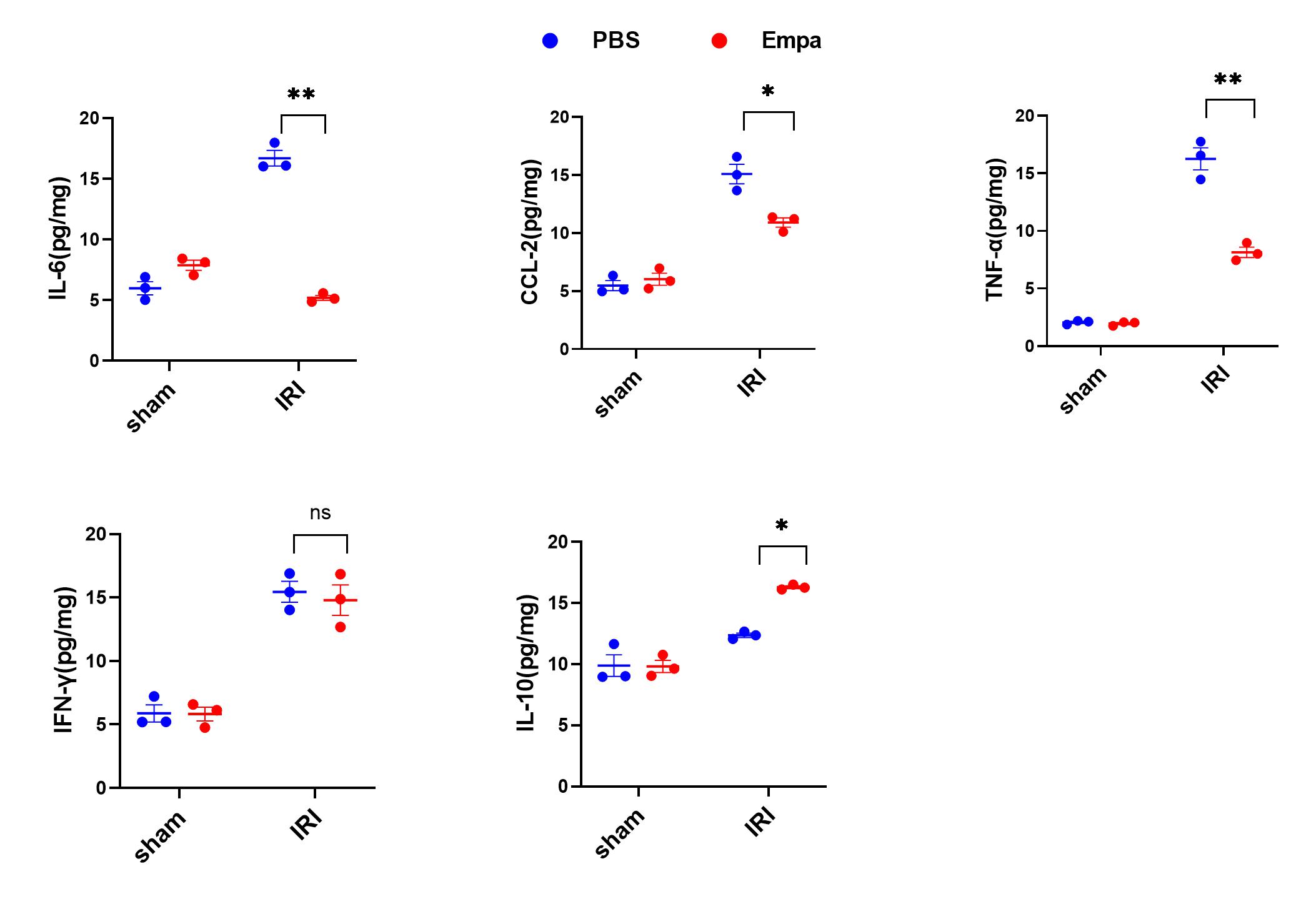
**

**Fig.S4 Detection of inflammatory response in kidney tissue during IRI** Tail vein injection of Empagliflozin (10 mg/kg/d) was performed 12 hours prior to renal IRI modeling, and blood was collected from the orbital venous plexus on 5-7 days. ELISA was performed to detect the expression levels of peripheral blood pro-inflammatory factors IL-6, CCL-2, TNF-α, IFN-γ and the expression level of anti-inflammatory factor IL-10. The experiments were repeated independently at least three times, and the data were presented as the means±SEM. Statistical significance with respect to control was marked with *P < 0.05, **P < 0.01 or ***P < 0.001 respectively.

**
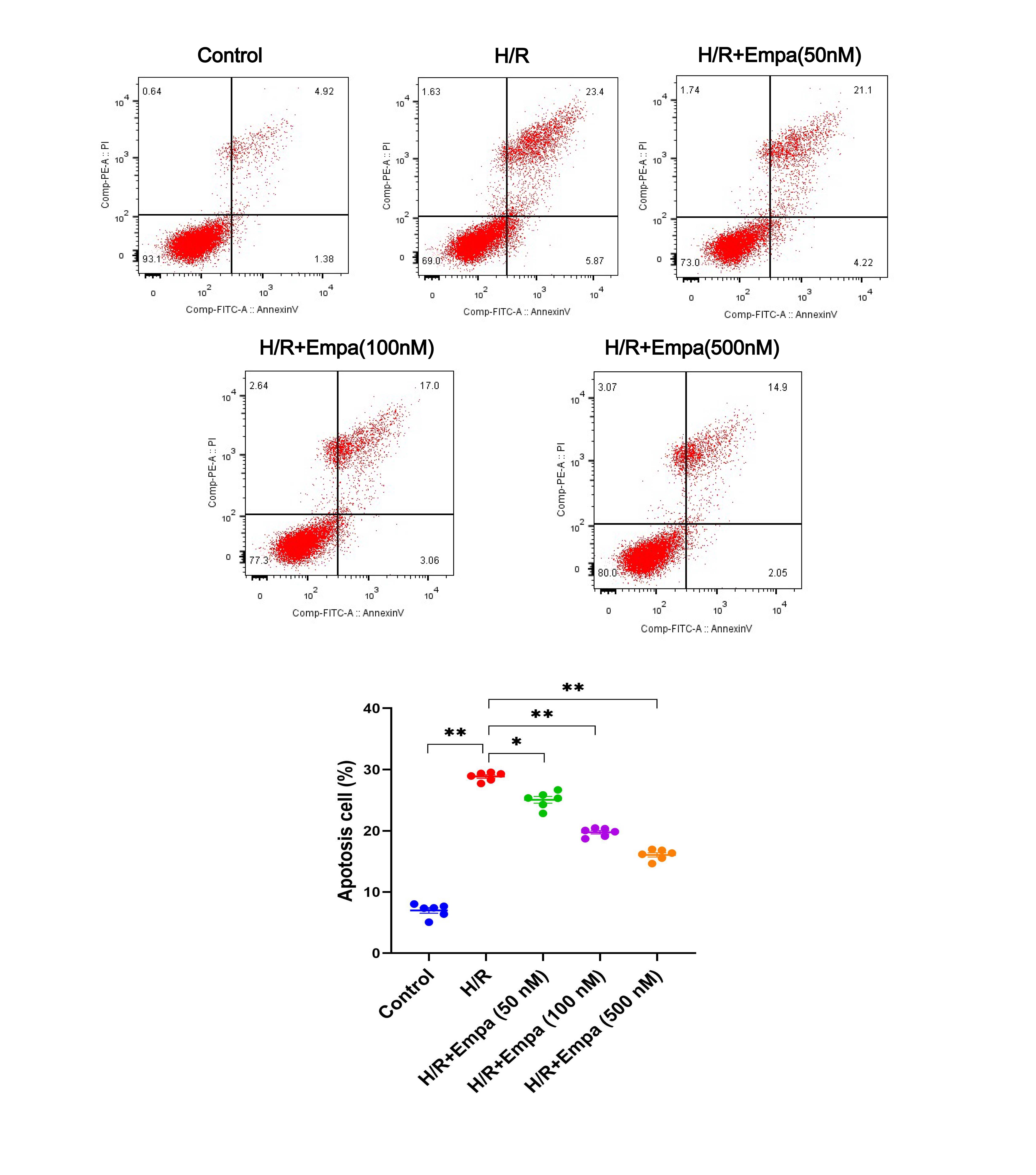
**

**Fig.S5 Analysis of the appropriate dose of Empagliflozin by flow cytometry** Apoptosis was examined through flow cytometry and CCK8 assay. Four experimental groups and control: H/R, H/R+Empagliflozin(50nM), H/R+Empagliflozin(100nM), H/R+ Empagliflozin(500nM) were set up to treat renal tubular epithelial cells. Then, results were quantified to obtain apoptotic proportion. The experiments were repeated independently at least three times, and the data were presented as the means±SEM. Statistical significance with respect to control was marked with *P < 0.05, **P < 0.01 or ***P < 0.001 respectively.
